# Beyond the template: the needs of tribal women and their experiences with maternity services in Odisha, India

**DOI:** 10.1186/s12939-018-0850-9

**Published:** 2018-09-24

**Authors:** Sana Q. Contractor, Abhijit Das, Jashodhara Dasgupta, Sara Van Belle

**Affiliations:** 1 Centre for Health and Social Justice, Basement of Young Women’s Hostel No. 2, Avenue 21, G Block, Saket, New Delhi, Delhi 110017 India; 2 SAHAYOG, A-240 Indira Nagar, Lucknow, Uttar Pradesh 226016 India; 3 Department of Public Health, Institute of Tropical Medicine, Nationalestraat 155, 2000 Antwerp, Belgium

## Abstract

**Background:**

Over the past 15 years, several efforts have been made by the Government of India to improve maternal health, primarily through providing cash incentives to increase institutional child birth and strengthen services in the public health system. The result has been a definite but unequal increase in the proportion of institutional deliveries, across geographical areas and social groups. Tribal (indigenous) communities are one such group in which the proportion of institutional deliveries is low. The persistence of these inequities indicates that a different approach is required to address the maternal health challenges in these communities.

**Methods:**

This paper describes an exploratory study in Rayagada District of Odisha which aimed to understand tribal women’s experiences with pregnancy and childbirth and their interactions with the formal health system. Methods included in-depth interviews with women, traditional healers and formal health care providers and outreach workers, observations in the community and health facilities.

**Results:**

The exploration of traditional practices shows that in this community, pregnancy and childbirth is treated as part of a natural process, not requiring external intervention. There is a well-established practice of birthing in the community which also recognizes the need for health system interventions in case of high-risk births or complications. However, there has been no effort by the health system to build on this traditional understanding of safety of woman and child. Instead, the system continues to rely on incentives and disincentives to motivate women. Traditional health providers who are important stakeholders have not been integrated into the health system. Despite the immense difficulties that women face, however, they do access health facilities, but barriers of distance, language, cultural inappropriateness of services, and experiences of gross violations have further compounded their distrust.

**Conclusions:**

The results of the study suggest a re-examining of the very approach to addressing maternal health in this community. The study calls for reorienting maternal health services, to be responsive to the requirements of tribal women, cater to their cultural needs, provide support to domiciliary deliveries, invest in building trust with the community, and preserve beneficial traditional practices.

## Background

India has made significant strides in reducing maternal mortality, with the maternal mortality ratio (MMR) declining from 254 in 2004–06 [1] to 167 in 2011–2013 [2]. Considerable policy attention was given to maternal health in the Millenium Development Goal (MDG) era, including the introduction of a national conditional cash transfer scheme to incentivize institutional deliveries - the *Janani Suraksha Yojana* (JSY) - in 2005. Health facilities in rural areas were strengthened through the National Rural Health Mission (NRHM) and a cadre of a community health volunteers called Accredited Social Health Activists (ASHAs) were introduced to support and encourage pregnant women to deliver in public health facilities. *Janani Shishu Suraksha Karyakram* (JSSK, or mother and child safety programme) was launched in 2011 to address high out of pocket expenses which were perceived as a key barrier to (skilled) institutional delivery.

**Fig. 1:**
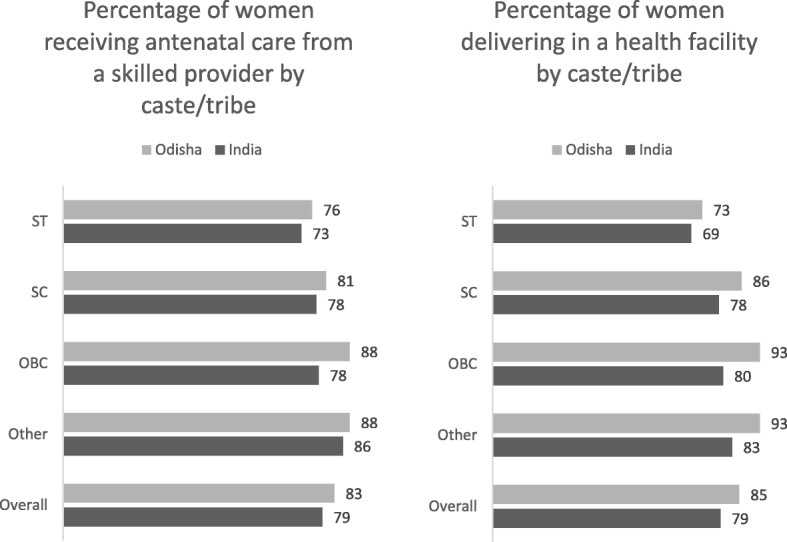
Coverage of antenatal care and institutional childbirth by caste, India and Odisha, Source: National Family Health Survey (NFHS-4), 2015–16: India. Mumbai: IIPS. 2017 and National Family Health Survey (NFHS-4), 2015–16: Odisha. Mumbai: IIPS. 2017.

As a result of these initiatives, the utilization of maternal health care as well as institutional deliveries increased dramatically. The percentage of mothers who had at least four antenatal care visits increased from 37% in 2005–06 to 52% in 2015–16 and the proportion of institutional births increased from 39% in 2005–06 to 79% in 2015–16 [3]. However this overall improvement masks inequities across geographic and socioeconomic groups (Fig. 1). States such as Uttar Pradesh and Assam have an MMR close to 300 [2]. Kerala and Tamil Nadu have almost 100% institutional delivery while only a third of births in Nagaland take place in institutions [3]. Successive rounds of the Annual Health Surveys (2010–11 and 2011–12) showed that 207 out of the 284 high focus districts remained in the same range of MMR [4].

A recent review of data from nine states shows that although inequalities in access to institutional delivery have reduced after JSY was introduced, the poorest administrative divisions had 135 more maternal deaths per 100,000 live births as compared to the richest divisions, and MMR declined 4 times faster in the richest divisions compared to the poorest ones [5].

There is a growing recognition at the global level that, while overall improvement in maternal health indicators has taken place, inequities exist within countries [6]. The sustainable development goals (SDGs) seek to address these inequities, reflected in their slogan, “Leave no one behind”. While there is interest in reducing inequities, the widespread assumption is that the interventions themselves are appropriate and implementation bottlenecks are the problem; therefore, previous research has recommended “improved targeting” of disadvantaged and marginalized groups. (See for instance Lim et al. 2010 [7]). However, such an approach based on increasing coverage and availability of services through targeting is inappropriate and insufficient to address inequities. Childbirth is an inherently cultural event and research from different parts of the world shows that maternal and neonatal care practices are deeply related to socio-cultural beliefs (see for instance, Winch et al. 2005 [8] from Bangladesh, Morris et al. 2014 [9] from Madagascar, Lori et al. 2011 [10] from Liberia). Diversities in histories, topographies, health system capabilities and relationships between communities and health systems cannot be ignored if interventions are to be successful [11]. A comprehensive contextual analysis of cultural, political and social factors is required to understand the root causes of inequities and propose localized solutions.

This paper explores the experiences with maternal health services of women from a marginalized group in India – one of the tribal communities. Tribal communities, or Scheduled Tribes (STs) as they are known, are historically disadvantaged indigenous communities (Constitution of India (Scheduled Tribes, ST) Order, 1950). Among this population, the proportion of home deliveries continues to be comparatively higher than the general population, despite the incentives provided by JSY to draw women to health facilities. Tribal women are less likely to receive antenatal care from a skilled provider (73% as compared to 86% from other castes), less likely to deliver in a health facility (68% as compared to 83% from other castes) and less likely to receive a post-natal check-up within the first 2 days of birth (59% as against 69% from other castes) [3]. A UNICEF study found that both Schedule Caste and Schedule Tribe women make up a disproportionately large proportion of maternal deaths in some states [12]. A number of studies have documented poor access to quality maternal health services in districts with higher tribal populations. For instance, an investigation of maternal deaths in Barwani, Madhya Pradesh, (which a high proportion of tribal population), found an absence of antenatal care, lack of skilled birth attendants and poor emergency obstetric care [13]. An investigation in Godda district of Jharkhand (also predominantly tribal) found significant gaps in the health system response to maternal complications and low institutional birth rates among ST women [14].

The persistence of these inequalities indicates a need to explore the cultural, social and economic barriers that affect tribal women’s access to government maternal care as delivered today. This study, therefore, aims to explore: 1) tribal women’s perceptions and practices related to childbirth and pregnancy; 2) pregnant women’s experiences with government maternal health schemes and services.

### Study setting

The study is located in the state of Odisha, where 22% of the population is ST, as compared to 8.6% of the total population of India. Odisha is also one of the High Focus states for improving maternal health, with an MMR is of 222 deaths per 100,000 births, compared to India’s 167 deaths for India [2]. Tribal women in Odisha are 2.5 times more likely to bear a child by age 19 years, and 2.7 times more likely to have more than four children. Tribal mothers are 1.3 times more likely to be underweight and anemic [15]. While 85% of all women in Odisha deliver in health facilities, only 73% of tribal women deliver in health facilities [16].

Maternal mortality is a high priority issue for the Odisha state government and in addition to JSY, and JSSK, the state government introduced the MAMATA scheme, to “promote health seeking behavior” and provide wage compensation and improved nutrition to pregnant and lactating women. Launched in 2010, MAMATA provides up to Rs 5000 (USD 80) for antenatal care and infant care services, in addition to JSY, which provides an incentive for institutional childbirth. The MAMTA incentive is given in four installments over pregnancy and post-partum (until the child is 9 months old) to ensure that mothers access a range of maternal and child health services.

The results have been quite dramatic. While only 36% of women in Odisha delivered in institutions in 2005–2006, this figure more than doubled to 85% in 2015–16 [16]. Yet, tribal communities are disadvantaged. The tribal dominated districts in the Southern Division record an MMR of 245**.**

This study was conducted in Kalyansinghpur block of the mineral rich and densely forested district of Rayagada in southern Odisha. STs comprise 56% of the population of Rayagada. [17]. In terms of development indicators, Rayagada district is one of the most deprived districts in Odisha [17]. Only 72% of births take place in institutions, compared to 85% for the state [3]. Kalyansinghpur block has a population of 66,000 of which 65% is tribal, mostly belonging to the *Kondha* tribe [18]. The block has a literacy rate of 38%, and is one of the poorest blocks in the district. The study was limited to 9 villages situated in one Gram Panchayat (population 4663). Of the nine villages, four were easily accessible and connected by all-weather roads, while five were isolated and located in the hills. Four out of the five isolated villages did not have any all-weather roads; one did, but people had to walk at least 5 km to reach a vehicle. The isolated villages were materially more deprived than the accessible ones. While villages with more accessibility had hand pumps for water, the isolated ones depended on river streams. In two villages, people walked close to 6 km, crossing hills and streams, just in order to get food rations from the nearest distribution center. This provided us an opportunity to explore the differences in birthing customs and utilization of formal maternal health services, based on the relative isolation of the village.

### The people

The *Kondhs* are forest and hill dwelling communities and are culturally, socially and linguistically different from the mainstream Odia population [19]. Anthropological studies describe three kinds of *Kondhs* – the *Dongaria* (or hill dwelling), the *Desia* (or plain dwelling) and the *Kuttia Kondh*. [20]. This study is situated among the plain *Kondh*s. There is also a history of conflict between the *Dongaria Kondh* community, the state and mining corporations in the region [21]. The Niyamgiri hill, where the local *Dongaria Kondh* tribe has fiercely protested against acquisition by Vedanta, a mining conglomerate [21], is located adjacent to the area where this study was conducted. The hill has spiritual value for the people and they depend on it for produce and herbs.

*Kondh* society is patrilineal and patrilocal; polygyny is not uncommon [20]. Most women in our study sample reported having 4 to 5 children, with first delivery occurring between 16 and 20 years of age. In addition to multi-parity and low age at first pregnancy, poor nutrition and lack of rest during pregnancy also jeopardizes maternal health outcomes. In our study area, women ate only two meals a day consisting of rice, roasted wheat flour (*sattu*) or a watery porridge made of millets (*ragi*), the latter being part of the traditional diet [22].

Despite tribal women having more independence and fewer restrictions than women belonging to caste Hindu communities [23], their status remained low and decision-making regarding issues like seeking health care and large expenditures rested with men. Women tended fields, reared animals, and sold produce in addition to housekeeping, fetching water and firewood, cooking and caring for children. This workload carried into pregnancy. Women explained that strenuous labour could not be avoided as the family’s livelihood depended on it.

### Health service provision in the field area

The health facilities serving the field area included a sub-centre, a primary health centre, a community health centre and the district hospital. A village health and nutrition day was held each month at the village level, which was conducted by the Auxillary Nurse-Midwife (ANM). The community’s closest contact with the health system were the ASHA community health workers. There were 5 ASHAs in the field area, some of who were responsible for taking care of 3–4 villages. The ASHA belonged to the village, but was not from the tribal community. There was also an Anganwadi Worker (crèche/pre-school worker) (AWW) in the villages who provided nutrition services to mothers and children.

The sub-centre serving the community was usually shut as the ANM, who should have been residing at the centre, spent her time travelling across 15 villages in her duty area to provide outreach services, and was rarely available at the centre. The primary health centre, which should ideally have been conducting normal deliveries as well as providing basic emergency obstetric care, was ill-equipped and poorly staffed, and no delivery services were being conducted. The closest delivery point was a nine-bedded community health centre located about 15 kms from the villages, which conducted only normal deliveries. The community health centre building was run down and there was a dearth of water and electricity. In the warmer months, women had to move out to the verandah to escape the heat. Even though a community health centre is expected to provide comprehensive emergency obstetric care, it did not deliver this care due to the lack of skilled providers and non-availability of life-saving commodities like blood. In case of complications where women were severely anaemic or had obstructed labour or eclampsia, they were referred to the district hospital. The district hospital was able to manage most emergencies, but in case it could not, women were referred to a private missionary hospital about 50 km away.

## Methods

The study used four qualitative methods, which provided different perspectives on the questions being explored (Table 1). Unstructured group discussions explored community perceptions around pregnancy and childbirth, and issues around access and quality of public health facilities. In-depth interviews explored women’s actual experiences and practices around their own pregnancy and childbirth. Key informant interviews with health service providers yielded contextual information about the field area. Observations allowed the researcher to triangulate the information and also gain first-hand information especially about the status of health facilities. The methods complemented one another and allowed us to triangulate information as well as explore questions in-depth, and from different perspectives. The tools were open-ended and laid out broad areas of exploration. Sample probes were provided to the field investigator, however these were only meant to aid the interviews/group discussions/observations, rather than serve as actual questions.

1. **In-depth interviews (*n*** **= 36)**: The sample size was not pre-determined, but rather evolved based on what emerged in field work. The sampling for interviews was purposive. Women who had delivered recently or who were currently pregnant were identified through the ANM and ASHA. None refused to participate in the study. Interviews took women through the process of their pregnancy asking for a detailed recounting of the antenatal, delivery and post-natal period. Prompts such as “Can you tell me what your average day was like during pregnancy?” “What happened when you went for a checkup” etc. Interviews typically lasted 40–50 min and were carried out in a private space (typically the house of the woman) to ensure confidentiality. Interviews were usually done in the afternoons when women were alone and had minimal interruptions.
2. **Key informant interviews (*n*** **= 9):** Key informant interviews with formal service providers and former Traditional Birth Attendants (Dais) explored the health services available and challenges to offering locally tailored maternity care services.
3. **Participant observations (*n*** **= 5)**: Researchers observed Village Health and Nutrition Days where outreach services are provided, and interactions of women with health care providers in the Community Health Center. Health facility observations were also conducted to assess their conditions with respect to infrastructure and services. Checklists were used during observation, which asked researchers to observe for example the available facilities, infrastructure, cleanliness and upkeep, who all were in the facility, and so on.
4. **Group Discussions (*n*** **= 3)**: Semi-structured group discussions were carried out with women in three villages. The groups consisted of 5–8 women of different age groups, all belonging to the *Kondh* community, selected by convenience. Group discussions were carried out in a community space where meetings of local self-help groups and other community meetings take place. When discussions began, often others from the village would gather as well. However after the initial conversation regarding general information about the village and community, they would leave and more sensitive issues related to pregnancy and reproduction could be discussed. It was also difficult to maintain a static group as women would repeatedly get engaged in other work, hence structured group discussions were not possible. Group discussions typically lasted for about one hour in all.
5. **Field notes:** Extensive field notes were taken during observations, interviews and discussions. At the health facilities, these also recorded the woman’s movement through the facility and communication between the woman and providers.

**Table 1:** Methods used for data collection

Method	Number
In-depth interviews	36
Recently delivered women in nine study villages	15
• Home delivery	(10)
• Institutional delivery	(5)
Pregnant women in nine study villages	7
Kondh women visiting health facilities for delivery	12
• Community health centre	(10)
• District hospital	(2)
Husbands of women who died of maternity-related causes in nearby panchayats	2
Key informant interviews	9
ASHA	2
AWW	2
ANM	2
Maternity-waiting home (MaaGhar) co-ordinator	1
Former Traditional Birth Attendants (Dais)	2
Observations	5
Village Health and Nutrition Days	2
Primary Health Centre	1
Community Health Centre	1
Group discussions (each with 5 to 8 women)	3

### Methodology

The study was exploratory, using qualitative methods to understand the culture, practices and perspectives around maternal health in the community. Fieldwork was conducted between December 2014 and April 2015 in collaboration with a local non-profit organization^1^ that works on livelihood issues. The NGO had been working in the field for a long period and has a thorough knowledge of the social dynamics within the communities. The local NGO facilitated the fieldwork, helped identify participants, and was able to ensure that women and health workers were willing to be interviewed for the study. An experienced field investigator, familiar with the maternal health programme in the state and who spoke Odia (the state language), was stationed in the field for one and a half months. She was accompanied by a local assistant who spoke *Kui*^2^ (the tribal language) and provided translation support. The first author is a researcher with a master’s degree in public health. She made three visits to the field during the time of the study – twice for conducting interviews with key informants and once to discuss and validate the emerging findings with local organizations.

### Data analysis

All data was collected in Odia, but transcribed in English by the field investigator. Transcripts were reviewed by the first author and gaps/new areas of exploration were identified which warranted further fieldwork. The overall analytical approach was grounded theory. Grounded theory is used to make sense of perceptions of participants about certain events or phenomena, rather than being informed by a pre-existing hypothesis [24]. It is being used in health systems research to explore patients’ perceptions and experiences of services as well as of illness [25]. In the early stages of data collection, the data were first open-coded by the first author. These were then reviewed with the field investigator as the data collection and analysis proceeded, and the lack of congruence between the community’s perceptions around pregnancy and childbirth and the health system’s approach began to emerge as an important finding. Subsequent data collection, especially observations, conducted after the interviews, explored this aspect more deeply. Once data collection was complete, themes were identified by the first author. These were reviewed by the research team and finalized.

### Ethical concerns

Written consent (via a signature or thumb print) of all respondents was obtained on a consent form in Odia that was read out to the respondents. The consent form provided women with information about the purpose of the study, contact details of the researchers, assured confidentiality and informed them of their rights to stop the interview at any time. In case the respondent did not speak Odia, the consent form was orally translated into *Kui*. The study methodology was reviewed by an expert committee consisting of researchers and practitioners working on tribal health in the Indian context. The study design and tools were piloted by the first author, and reviewed by the committee. The findings of the study were shared with the local organization, for dissemination to the community, and with local NGOs with the purpose of validation. The final research report was shared with policy makers including groups exploring policy making for tribal communities in India.

## Results

Over the past decade, the government of India has adopted a model that prescribes institutional childbirth for all deliveries, and this has been promoted through the conditional cash transfer scheme of JSY [4]. Limited success with previous supply–side interventions in raising the proportions of skilled attendance at births and the growing evidence on the effectiveness of demand–side financing schemes were important factors that led to the implementation of JSY [26]. While the scheme has persuaded women to begin accessing health facilities for antenatal services and delivery care, we find that the health system has not been able to adapt to women’s needs. In the following sections, we explore the areas of dissonance between the health system and the realities of tribal women, and the ways in which this impacts their health and well-being during pregnancy and childbirth.

### The tribal approach to childbirth as a normal event

In contrast to the government’s promotion of institutional delivery guided by the belief that every childbirth could potentially lead to complications, we found that in this community pregnancy and childbirth is perceived as a natural process, not requiring much external intervention. Traditional practices both in the antenatal and post-natal period are primarily geared towards protecting the mother and child, through conducting prayer ceremonies to ward off evil spirits, and restricting diet. Little importance is given to ‘small problems’ like fever in the post-natal period or swelling of feet in the antenatal period as these are considered a common part of pregnancy. Women go through childbirth without disturbance to their regular life. The woman can go about her daily activities and work until the time of delivery and this is an important consideration for them. The delivery is conducted in the home compound with a close family member as an attendant, in a surrounding familiar to the woman. The process of delivery is said to be ‘impure’ and so delivery takes place outside the house. It takes place in a squatting position and women are allowed to walk around in between contractions. Generally, no herbs are given during pregnancy or labour. After delivery, the placenta is buried in a pit and covered with sticks. The woman bathes on the pit to wash away all the impurity, and then it is filled up. The purpose of this ritual, as reported by women, was to protect the baby. If the placenta is left out in the open it is likely that it would be eaten by animals and this could bring harm to the baby. Along with this understanding of pregnancy and childbirth as a primarily natural process, there is awareness that pregnancy can get complicated. The traditional birth attendant for instance mentioned that in case of prolonged labour, retained placenta or unrestrained bleeding, it is necessary to take the woman to the health facility. This suggests that there is a well-established practice of birthing in the community, which includes some rituals as safety precautions and also recognizes the need for health system interventions in certain cases.

### Persistence and neglect of home births

As discussed earlier, the thrust of maternal health policies over the past decade has been on institutionalizing childbirth and this has led to a corresponding increase in the proportion of women giving birth in public health facilities [26]. This was also true in our field area, but despite the increase, 26 out of the 70 deliveries that were recorded in the year preceding the study took place at home (as mentioned in the ANM’s records). It was interesting to note that home deliveries took place both in villages that were well connected by roads as well as those without connectivity. This suggests that geographical isolation is not the only factor that prevents women from utilizing the formal health system for delivery care. Women gave different reasons for delivering at home. Since the burden of both household work and livelihood was borne by women, they were concerned about the number of days that would be lost if they went to the hospital. Birthing at home meant that they could go back to work immediately and tend to their children. One woman who had had four previous normal births at home, felt that no additional advantage was provided by going to the hospital. In fact, it only meant loss of wages and additional expenses. So she was planning to have her fifth birth at home as well.

Government policy stipulates that home deliveries be attended by a skilled birth attendant or a trained birth provider. JSY prescribes cash assistance of 500 rupees [approximately USD 7] for home birth as long as the pregnant women is below the poverty line and above 19 years of age, for up to two births [27]. In case a woman opts for a home birth, the ANM is expected to attend to the birth, and this has been laid out formally in her roles. The role of ANMs in practice however remains restricted to preventive services and providing antenatal care [28]. Significantly, none of the deliveries at home were attended by a skilled birth attendant. The delivery was usually attended to by a close family member, usually the mother in law, or an elderly woman in the household. The delivery was perceived to be complicated if labour went on for more than 12 h, or if there was excessive bleeding following delivery. In this situation, the woman was taken by the family to the community health centre. The traditional midwife or *Dai*, who used to attend home deliveries, had stopped intervening in cases of delivery for the past 10 years. She mentioned that now the focus was on taking women to health centers, for which ASHAs were motivating women, and so there was no role for her. However she narrated that in the past, she would attend to births and also called on the ANM’s help in some cases when she anticipated complications, such as when twins were expected or when the position of the baby was incorrect. This suggests that even when *Dais* were assisting births, there was a system by which they identified complications and sought help from skilled providers. There was no hesitation in sending women to the hospital when necessary. However this collaborative way of functioning does not exist anymore. As of today, there is no *Dai* to help women during birth, nor does the ANM assist home births. As a result, women who deliver at home are left with no skilled support.

### A preoccupation with numbers as a measure of performance

It was evident that health care providers, especially ASHAs and ANMs, were very concerned with ensuring that all women delivered in institutions. We noted that not all deliveries that were recorded as ‘institutional delivery’ were necessarily conducted in a public health facility. Several women who actually delivered at home were later taken to the health facility and recorded as institutional deliveries so that they could avail of the JSY incentive. The outreach workers acknowledged that it was difficult to get women to come for institutional deliveries. They genuinely believed that they were working for the benefit of the laboring woman by encouraging her go to the health facility but, according to them, women were resistant. The following case study narrated by an Anganwadi worker illustrates this impression of the health workers. It is also an example of how women who deliver at home eventually end up being recorded as institutional deliveries:

She was not interested in going to the hospital for delivery. They say, “We are Adivasi people, we don't want to go outside and other male member shouldn't touch us.” I told her, “We are all present for your benefit why shouldn't you listen to us? Both mother and baby will be safe in the health facility.” When she was not convinced I spoke to her husband, “If anything happens the family will suffer.” I thought I had convinced them and was feeling very happy. But then she ended up delivering at home. Her labour pains began in the morning but without informing anybody she went to the field for work. Only after she came back home she called me and asked me to contact the ASHA for the vehicle. In the meantime she delivered the baby before the vehicle reached the village. Thankfully her family agreed to visit the hospital and the cord was cut there. She received the Rs.1400/- for institutional delivery. However, many a times, if the vehicle reaches after they delivered, they refuse to visit the hospital. What's wrong with it, they can get treatment, child immunization and the cash benefit. (Interview with AWW)

There was a preoccupation with ensuring “coverage” of services for every single pregnant woman, and apart from fudging data (showing home births to be institutional births, as described above), outreach workers also used coercive tactics to achieve this. In order to get women to come to the VHND, the ANMs and AWWs at the local level instituted their own conditionalities: *“*We threaten them that if they do not come, they will not get their take-home ration. We know this is not true, but they believe us. What can we do, we will be in trouble if they don’t come” (interview with ANM).

### Poor communication resulting in ineffective antenatal care services

While there was a reliance on incentives and disincentives to increase coverage of services, there appeared to be a gap between the intended purpose of services and women’s understanding of it. During ANC check-ups, which were conducted at the VHND once a month, almost all women reported receiving iron folic acid (IFA) tablets, having an abdominal check-up, haemoglobin tested and blood pressure recorded. However, during observation of the VHND we observed that none of the women were explained what was being done and with what purpose. Women therefore did not understand why the tests were being done during antenatal check-ups and often they would not follow advice given. Although IFA tablets were provided to almost every woman, not a single woman had taken the full course. One reason for this could be that traditionally no medicinal herbs were taken during pregnancy for fear of harming the fetus and so women did not consider it proper to take any other form of medicines. One woman reported that she had experienced nausea and vomiting after taking the IFA tablet and so she discontinued it. Both, “cultural beliefs against consumption of medications during pregnancy” and “negative side effects” have been reported as barriers in IFA tablet consumption in previous research [29].

Women also did not give much importance to birth planning and preparedness and neither was this reinforced during antenatal visits. According to traditional knowledge, there was no concept of an expected date of delivery. When asked when the baby was due, a women said: “How can we predict when the baby will be born? It will be born when it is time.” There was also a belief that complications and death, if they have to occur, will occur anyway and nothing can really be done to stop them apart from allaying the spirit (*doomba)*. Therefore, the need for identification of high risk women or birth planning was not something that women were able to appreciate. Women’s interactions with outreach workers also did not address this set of beliefs. Thus, although the coverage of antenatal care was good, it did not locate itself within women’s concerns. Instead of building on the well-established local understanding of safety during pregnancy the approach was to ignore these and introduce new practices, the reasons for which were not clear to the women.

### Barrier of distance and inadequacy of transport services

Tribal communities in the state of Odisha typically reside in forest and hilly areas, which are geographically challenging to reach. In light of this, the Odisha Government has made available a free and dedicated ambulance service. However, women in the more isolated villages reported that access to a vehicle was a problem. In these areas, women had to be brought quite a distance to the motorable road in order to reach the ambulance.

A is an isolated village with no government services at all. There is no ASHA or AWW situated in the village, nor do they visit. All deliveries occur at home. The reason for this is quite obvious, considering the long and difficult journey that a woman would have to undertake to get from the village to the CHC. A woman from A would first have to climb down for about 6 kms in a rough hilly terrain crossing four streams, to village B. From here she would need to walk downhill about 5 kms to village C and then another 1.5-2 km to D, after crossing two streams. For this entire journey there is no road. From D to E there is a dirt road of about 2.5-3 km. Only once the woman reaches E does she have access to an all-weather motorable road where an ambulance can be reached to get to the CHC which is located about 12 km away. The entire journey takes around 6 hours. (Researcher’s field notes)

Almost everyone – respondents, families and services providers – reported that there were problems in reaching the ambulance. To begin with, most villages had very poor cellular phone connectivity. When cellular phone connectivity was available, the emergency helpline for ambulances was perpetually busy. Even when the ambulance was contacted, it could take as long as 4 h for it to reach the village. In case of isolated villages, ambulances refused outright. Of the 12 women who we observed at the community health centre, 4 had come from far-off distances and had not been able to come by ambulance.

Recognizing that geographic isolation is a challenge, maternity waiting homes have been set up by the Government of Odisha since 2012, where women can stay for approximately one month before delivery. Women who are identified as high risk in the antenatal period are referred to a waiting home and transport is also provided. However, our field observations indicate that tribal women were not using the waiting homes and most people in the community were unaware of their existence. Women who had been referred to a waiting home were not aware of its purpose and were concerned about out-of-pocket expenses as well as neglect of domestic responsibilities, and therefore refused to stay there.

### Appropriateness of health service delivery: Cultural and linguistic barriers

While a growing number of women are accessing health facilities for childbirth, their experiences in these facilities were marred by a number of issues, one of which was the alienating environment of the health facility. Language presented itself as a key barrier between health care providers and the women. Because most of the women spoke *Kui*, all communication with the health care providers was directed through the ASHA. Women found this very unnerving as they were unaware of what was happening around them.

S, a 16 year old first time mother, was sent to the District Hospital in Rayagada for delivery. S was very young, weak and severely anaemic, hence the ANM had recommended that she be taken to the institution before she went into labour. On reaching Singhpur CHC, she was referred to the District Hospital as a complicated case. S had to stay in the District hospital for 10 days before she delivered her child. The ASHA who had accompanied her could not stay for this entire period. She was required to stay alone (without her family, who waited outside) and did not understand the language that was being spoken. S says that she will never go back to the institution for delivery. (paraphrased interview with recently delivered woman).

The health care providers had also become accustomed to this situation and did not even attempt to communicate with the women. In one instance, a woman’s prescription carried the ASHA’s name rather than the woman’s. In another instance, a woman with fever during pregnancy was turned away from the CHC and asked to return with the ASHA (researcher’s fieldnotes).

Apart from language, there were other things about the health facility that did not agree with women, such as the food. Women were provided food that was insufficient as well as unfamiliar for them – *sooji* (semolina) in the morning, one slice of bread, a glass of milk and an egg in the afternoon, and one slice of bread and milk at night. The food, though commonplace, was unfamiliar and not in keeping with the dietary habits of the *Kondh* community. For example the *Kondhs* do not consume milk and so it was either discarded or returned.

The surroundings and birth practices were also unfamiliar to the woman. Delivery took place in the lying position as opposed to the squatting position that women were accustomed to. As one woman remarked in a group discussion:

[at the hospital]… all women lay in bed. In my case I didn’t want to lie in bed. They told me not to worry. If they allowed me to sit it would have been better. But they don’t allow that. I know their problem. If we sit how could they check? And it is not possible for the nurse to sit with every woman when the time comes for delivery. (Recently delivered woman in a group discussion).

The experiences of women emphasize that birthing in institutions is very different from women’s experiences of delivery at home. Accommodating women’s concerns requires structural adjustments be made in health facilities, however no efforts were being made to do so.

### Exclusion of ‘informal’ and ‘traditional’ service providers

Community members consult a range of traditional healers and informal (untrained) providers for various health issues, including during pregnancy. For routine illness, two local informal (untrained) providers in the area were consulted, but respondents insisted that informal providers play no role in handling childbirth. Despite this denial, in one case of post-natal complications, the family reported that they called the informal provider to give the mother an injection. This silence around the informal providers could be attributed to a circular from the district collector (a powerful local government administrator) forbidding informal providers from providing any services to pregnant women. While this circular may have been issued to avert harm, it appears that the practice has merely gone underground.

Among traditional healers, two important figures, the *Bejini* (or sorcerer) and the *Dai*, have significant roles to play. The *Bejini* is usually a single woman considered to have black magic powers. The *Bejini* is consulted in the antenatal period to predict the *doomba* (spirit) of the baby and such a prediction is said to be indicative of whether a complication is likely to occur. She usually accepts an animal sacrifice (often a hen) and conducts a ceremony to heal or avert a possible complication. The *Dai* is the most important traditional health care provider, however, her role has been made irrelevant. When the NRHM was introduced, the Dai mentioned that she had been considered for the post of ASHA and had also undergone training. However the role required her to travel to a number of hamlets, which are located far apart. This was something she found difficult and she therefore refused to become an ASHA. The frontline providers considered traditional providers to be negative influencers of women’s health. They recounted incidents where villagers had refused to accept formal health services on the advice of the traditional provider. However, despite the apparently important roles that informal and traditional providers have, the formal health system had no way of engaging them.

### Lack of trust due to adverse experiences and accountability failures

A significant factor that affected women’s decision whether to choose institutional care was that of others’ or their own adverse experiences. A few months before we began fieldwork, two maternal deaths had taken place in neighbouring villages. Whenever we asked women about institutional deliveries they cited these cases. In one of these cases, the woman had “run away” from the hospital and refused to return in the post-partum period when she was extremely ill.

B, a 24 year old pregnant woman with two previous institutional deliveries, was taken to the PHC at around 8 months because she developed blurred vision and severe headaches. The doctor told her husband that she had malaria and referred her to the CHC and from there to the District Hospital. For three days B stayed in the district hospital and got no relief. B’s husband was not allowed to stay by her side. Because B did not know the language, she could not communicate with any of the hospital staff. After three days of being ill, B and her husband left the hospital without informing anyone as she was not getting any better and they were getting no information. B felt that if she was going to die, she would prefer to die at home, in the presence of her family and children. They took a private vehicle and came back to B’s mother’s house where she delivered. The child did not survive. After the delivery, B was very weak and continued to have blurred vision. Her husband tried to convince her to go back to hospital, but she refused. Two days after the delivery, she died. A maternal death investigation was conducted which concluded that B had died because she had not adhered to medical treatment and left the hospital against medical advice. No effort was made to explore why B had left the hospital. (Interview with woman’s husband).

Such experiences of women with facilities easily spread to the entire community and they played a role in shaping women’s decisions about whether to go to the facility or not. For instance, in one case, a woman who delivered at the CHC but had a ‘weak child’ was asked to take the child to the district hospital, but she refused. Her neighbour’s child had been referred to the district hospital, they spent 20,000 rupees and eventually the baby died. Thus the family had no faith in the district hospital and felt that it would only lead to a loss of money and no relief. Moreover, there were women who had themselves faced bad experiences at the health facility and did not want to go back. One of the respondents had had a previous delivery in the CHC where she lost her child. In the next delivery she delayed informing the ASHA about her labour pains because she did not want to go to the institution. She did not want to take a risk a second time and found a way to deliver at home. This time the delivery was normal and the child was well.

To make matters worse, an instance was mentioned when doctors had attempted to a broker deal with the family of a deceased woman, in order to avoid blame. In one case of maternal death in a seemingly uncomplicated pregnancy, which was reported in the local media, health officials requested the family to tell the media that they were not at fault. According to the ASHA, *“*they promised to get the family the JSY benefits and the doctor who was in charge offered to pay the family 20,000 rupees for raising the children. The family accepted his offer. However after a few months, the doctor disappeared, and was not heard from again.” The woman’s husband felt cheated. He said:

Why should we go to hospitals when they do not care for us? If my wife had delivered at home she may have still died, but she would at least have received some food or water to drink. At the hospital she got nothing. If she had died at home, we would regret it, but at least she would be around us and we could have done something. We would not have to spend money to take her dead body back to village. What is the benefit for us in taking our women to hospital for delivery? (Interview with woman’s husband).

Perhaps because the woman belonged to the priest’s family, S’s story spread to other villages. No explanation was offered for her death by the system and in fact, the disappearance of the doctor deepened the sense of mistrust that people have in the system. In one informal interaction with women in a well-connected village, one of the older women remarked, “Why should they (our women) deliver in hospital? To die? If you have to die it should be with near and dear ones, where the person can have some food or water.”

## Discussion

Despite improvements in aggregate indicators of maternal health globally as well as in India, there is a growing recognition that underlying inequities within countries urgently need to be addressed. In the Indian context, there are gross inequities across geography as well as class and caste attributes. India’s Reproductive, Maternal, Neonatal, Child and Adolescent Health Policy 2013, also recognizes the importance of addressing inequities and acknowledges that several communities have been left behind in maternal health programming. However, with respect to tribal communities, it tends to view the problem as largely rooted in their geographic isolation, rather than their social and cultural exclusion ([30], page 54). A recent review of literature examining inequities in maternal and reproductive health from India identified five main social determinants as important to understanding inequities in the Indian context – Gender, Education, Age, Economic Status and Social Status (including Caste, Tribe and Religion) [31]. With respect to caste and tribe, the review found that although a few studies report caste/tribe based disparities in maternal health care utilization and outcomes, there is a dearth of qualitative studies that provide a contextual analysis of the reasons for inequities and barriers faced by marginalized communities. This study is an attempt to fill this gap, providing an insight into the interaction between a marginalized tribal community in Southern Odisha and the ambitious maternal health programs in the post-MDG era that have sought to promote skilled childbirth in order to reduce maternal mortality.

The study finds that in the community there is an established traditional understanding of safety during pregnancy and childbirth, along with an existing ecosystem of formal, informal and traditional health care providers who are seamlessly accessed by the community. However, the introduction of policies driving women to health facilities for delivery and increasing coverage of formal services, has sought to replace these rather than build on them, resulting in dissonances between the community’s reality and the nature of services that are provided by the health system. An examination of this dissonance provides guidance for both the implementation of maternal health programs, as well as for our understanding of inequities.

One of the most striking findings of this study is the mismatch between what tribal women want from maternal health services and what the health system provides, which reflects that public healthcare solutions are not adapted to or embedded in local contexts. In part, this is a result of top down, globally-driven policy making, not just in India but also in other countries where maternal health programming to institutionalize child births has been unsuited to local realities. Kvernflaten [32], for instance, describes the impact of the target oriented narrowing of the maternal health agenda to skilled birth attendance and institutional delivery in Nicaragua, and how this has resulted in stunting the role of community health workers and traditional birth attendants, and straining relationships between the community, the health workers and the health system. Tracing the shifts in global advocacy for improving maternal health, Storeng argues that there has been a growing influence of quantitative evidence in evidence-based advocacy in maternal health over the past two decades, which “reinforces an oversimplified “master-narrative” circumscribed by technical solutions to health problems” [33]. Freedman [11] points out that the implementation of “globally formulated standardized strategies” has masked the diversity of contexts and capabilities of health systems.

The present study similarly highlights the impact of target-setting and vertical programming that, in this case, has led to instances of coercion and falsification of data. Although these may be isolated instances, such practices signal the health system’s preoccupation with numbers, rather than responding to the needs of women. The use of carrot and stick approaches to rapidly increase service coverage disregards existing understandings of safety in pregnancy and childbirth in the community, and instead seeks to replace them with a new alien set of rules, which might not lead to sustainable improvement. The overwhelming focus on institutional childbirth has led to a neglect of women who deliver at home. This calls into question the ethical soundness of such a solution, especially when health facilities in many parts of India cannot provide high quality delivery services, referral services, and life-saving emergency care [4]. Maternal death reviews conducted by civil society organizations in India have found gross insufficiencies in maternal health services which lead to repeated referrals and cyclical delays in receiving care, ultimately leading to death [34, 35]. Rather than improve maternal health, such programmes may have contributed to further marginalizing the most marginalized groups, and worsened inequities in the longer term.

Addressing the needs of tribal communities in this context, therefore, requires not just better targeting of these groups, but rather, reorienting the interventions themselves. Providers are often frustrated by women’s reluctance to utilize health services, considering this reluctance to be caused by ignorance and low education. However, as Chapman found in her study on prenatal care seeking behavior among women in Mozambique, it is probably the service that needs to acknowledge and respond to women’s belief systems in order to be effective [36]. Similarly, in the context of this study, there is a case to be made for *culturally competent* health services that are able to communicate with women, cater to their food and language preferences, provide the option of delivering at home, or if delivery occurs in a health facility, enable squatting and allowing a birth companion to be present during delivery. Some of these interventions, such as having continuous support during labour, have also shown to improve outcomes for women [37].

There is growing evidence of service delivery innovations, health education models, participatory approaches and community based interventions that have helped make maternal health services more culturally appropriate [38]. In the Indian context, community based interventions with women’s groups, using the Participatory Learning and Action (PLA) methodology that builds on local knowledge and resources, have shown to have an impact on birth outcomes in tribal communities in the state of Jharkhand [39]. Similarly, in Pakistan community based maternal health interventions, which utilize local traditional birth attendants to identify risks and encourage referral to hospitals, have reduced perinatal deaths [40].

However, it would be simplistic to assume that merely tinkering with service delivery and outreach models will adequately address the needs of this community. Inequity is a result of structural exclusion and marginalization of certain communities, often perpetuated by the state. This exclusion results in a deficit of trust between the community and the public health system, which needs to be tackled. In the context of the present study, this lack of trust may be partially rooted in the fraught relationship between the ST community and the state, especially with regard to contestations over forest land and interests in mining in the region. This negative relationship was further exacerbated by instances of gross human rights violations, poor quality of care, and subsequent lack of accountability of health system actors, as is evident from the findings of this study. Research is increasingly recognizing the importance of trust-based health systems [41], and mending this relationship between the community and the health system through stronger public accountability measures is essential if trust is to be restored in the health system. This too is an important facet of inequities that must be addressed in a deliberate manner by health systems.

Finally, the findings of this study make a case for examining inequities beyond how coverage and outcome indicators vary across individual characteristics. While this analysis is an important starting point, there is need for deeper investigation into the causes of health-related inequities and the ways in which inequalities manifest in health settings. A recent synthesis of evidence [42] on health inequities in India finds that there is a predominant reliance of quantitative studies on large secondary data sets, which have not been collected with the express purpose of studying inequities. The study of inequities based on these data sets is restricted to outcomes and variables that are included in the data, leaving other parameters, especially those that cannot be quantified, out of the purview of investigation. In addition, relying on these data restricts analysis to a study of associations, rather than understanding how and why inequities persist. Qualitative methods have the potential to address this gap. Although they may be limited by the potential for generalizability, they have the potential to uncover not just how women’s specific disadvantages affect their health outcomes and access to services, but also how the health system, in the way that it designs health programs, might actually contribute to the exacerbation of inequities and marginalization of women.

## Conclusions

This study throws light on the experiences of tribal women with the formal health system, their lack of faith in the system and persistence of home deliveries despite the various incentives that are in place. Given the levels of impoverishment and destitution in the study community, it is no surprise that women are availing these incentives and the proportion of institutional deliveries is rising. However, it is important to reflect on whether a mere increase in childbirths conducted at the institution is a positive indicator in itself. These experiences suggest that there is a need for the health system to step back and reconsider its aggressive approach of institutionalizing deliveries. Action is required both in terms of strengthening the health system and addressing the physical and financial barriers to accessing maternal health services, as well as adapting health facilities to the needs of the community. This approach would truly improve maternal wellbeing, rather than using a carrot-and-stick approach of incentives and disincentives to get the community to utilize formal health services.

At the same time, there is a need to make some provision for those women who continue to deliver at home, especially in terms of provision of skilled birth attendance at home and swift access to emergency obstetric care in case of emergencies. In addition, there is a need for transparency, accountability and trust-building measures between the formal health system and the community. These measures can be established through platforms where the community can discuss their adverse experiences with the health system and can participate in designing and executing health programs. Sensitizing community outreach workers and health service providers to understand tribal customs and their unique problems will also serve to address the lack of trust between the providers and communities. Finally, the health system must find ways to cater to the specific cultural needs of tribal women during delivery (such as allowing women to choose a birthing position, allowing a birth companion, and so on) and build on their own existing systems, especially in terms of integration of traditional and informal providers.

## Footnotes

1 PRADAN (Professional Assistance for Development Action)

2 A Dravidian language that is similar in grammar to *Telugu* rather than *Odia*, the official state language.
